# Personal

**Published:** 1908-01

**Authors:** 


### PERSONAL.

CAPTAIN J. CARLISLE DEVRIES has been appointed national secretary of the Society of the Porto Rican Expedition during the Spanish War. Dr. DeVries's address is Watkins, N. Y., and those eligible for membership in the society may address him there. A surgeon in the National Guard of New York, Dr. DeVries entered the army as an assistant surgeon, accompanied General Miles to Porto Rico, and served as acting brigade surgeon with the rank of Major on the staff of Brigadier General O. H. Ernst. His reports of the work during the time of the expedition are of great interest and bring out many facts in connection with the diseases peculiar to the islands which have been of value to his successors.

DR. JANE W. CARROLL, of Buffalo, Supreme Medical Examiner of the Women's Catholic Benevolent Association, has recently issued a report giving a detailed statement of the work from June 15, 1904, to June 15, 1907, in which valuable statistics are given.

DR. BRANSFORD LEWIS, professor of genitourinary disease in the University of Saint Louis, will read a paper before the Buffalo Academy of Medicine, January 7, 1908. The subject of Dr. Lewis's dissertation is, "A discussion of practical cyctoscopy, with a presentation of examining, catheterising, and operative cystoscopes." Professor Lewis is one of the most prominent specialists in his chosen department of medicine residing in the middle west, and his reputation as an author and teacher is such as to justify the expectation of a large attendance at this meeting.

DR. KENNETH W. MILLICAN, of Chicago, was tendered a banquet at the Columbian Club, Saint Louis, Friday evening, November 8, 1907, which was attended by sixty of his professional friends. A number of excellent speeches were made and a gold watch was presented to Dr. Millican in token of the esteem in which he was held by the Saint Louis profession. The trend of feeling as developed by all the speeches was general regret at Dr. Millican's removal from Saint Louis to Chicago.

DR. DAVID E. WHEELER, of Buffalo, has removed from 525 to 391 Delaware Avenue. Hours: 2.00 to 3.00 and 7.30 to 8.30 P. M. except Saturday; Sunday, 2.00 to 6.00 P. M. Bell telephone, Tupper 1355.

